# Supplementary material for: Reanalysis of cluster randomised trial data to account for exposure misclassification using a per-protocol and complier-restricted approach
Source: Sci Rep. 2024 May 16;14:11207. doi: 10.1038/s41598-024-60896-9 (PMC11099120; doi:10.1038/s41598-024-60896-9)
Supplement: Supplementary file 1 — Supplementary Information. [file 41598_2024_60896_MOESM1_ESM.pdf]

Reanalysis of cluster randomised trial data to account for exposure misclassification using a per-protocol and complier-restricted approach

Supplemental Material

Suzanne M. Dufault, Stephanie K. Tanamas, Citra Indriani, Riris Andono Ahmad, Adi Utarini, Nicholas P. Jewell, Cameron P. Simmons, Katherine L. Anders

Contents

1 Human mobility **2**

2 Spatiotemporal Inverse Density Weighting **3**

3 Estimates of WEI **5**

4 Determining the "Compliers" for Complier-Restricted Analysis **7**

1 Human mobility

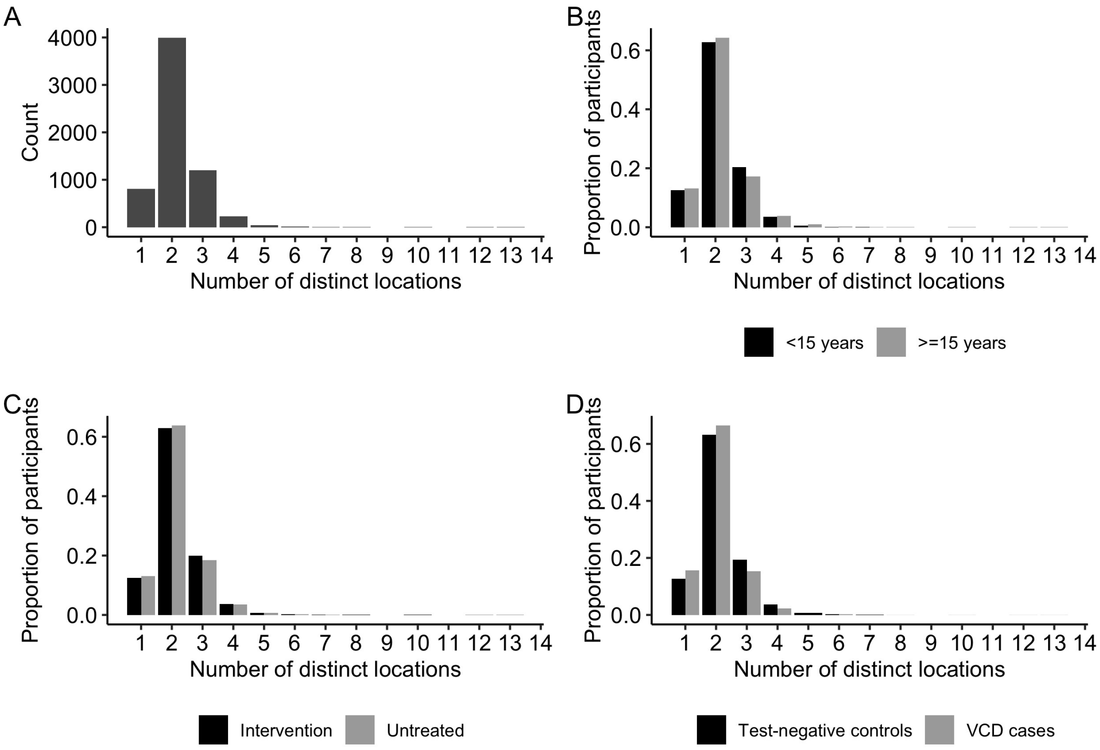

Figure S1: Number of distinct locations AWED trial participants self-reported visiting between 5am and 9pm during the 3-10 days prior to illness onset, including their primary residence. **A)** Frequency distribution of number of visited locations among 6306 participants in the analysis dataset. **B-D)** Relative frequency distribution of number of visited locations among participants <15 vs ≥ 15 years (B), participants resident in intervention vs untreated clusters (C), and virologically-confirmed dengue cases vs test-negative controls (D).

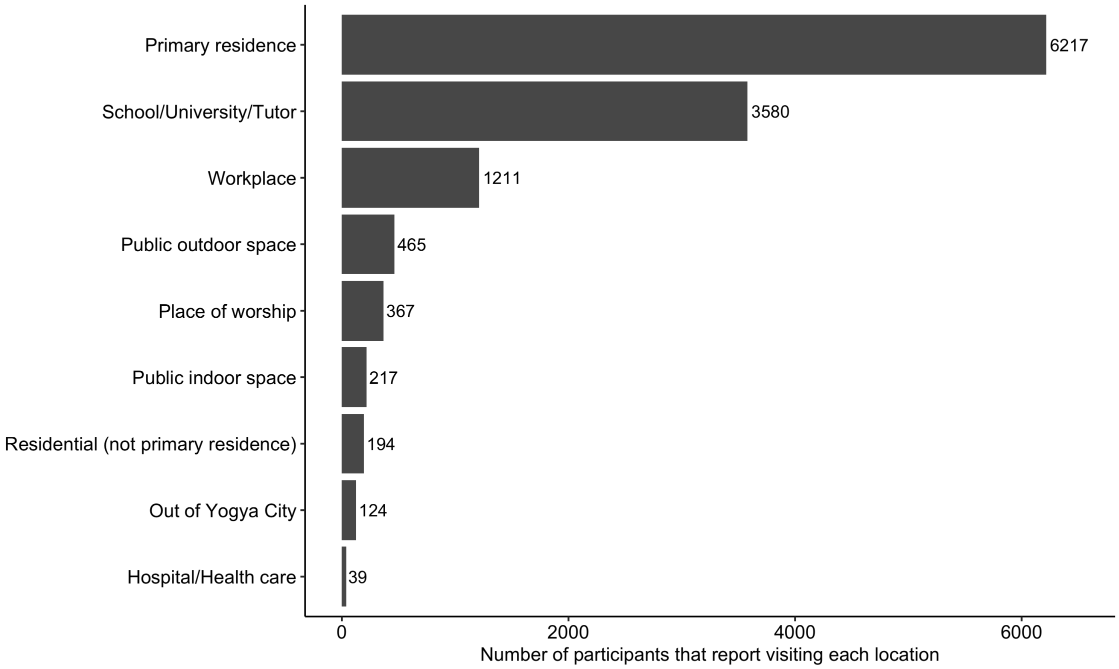

Figure S2: The top locations sorted by the number of participants who reported visiting each location.

2 Spatiotemporal Inverse Density Weighting

Before interpolating the *w*Mel surface across the study area, the highly variable trap-level proportions of *w*Mel mosquitoes were stabilised by summing the observed mosquito counts and observed mosquito counts with *w*Mel detected for the current and penultimate trap event. Spatiotemporal inverse density weighting (IDW) was used to construct interpolated *w*Mel surfaces based on this stabilised trap-level data.

The general formula for inverse density weighting (IDW) is given in Equation 1, where $\hat{Z}(\cdot)$ is the estimated proportion of mosquitoes with *w*Mel at a specified location s_0 and time t_0. Let s_i denote the ith observed trap location $\{s_i : i = 1, 2, \ldots, 455\}$ which has two spatial components (x_i, y_i), for instance, longitude and latitude. At each Let t_j denote the time of the jth observation where observations were made at $\{t_j : j = 1, 2, \ldots, T\}$.

$$\widehat{Z}(s_0; t_0) = \sum_{j=1}^{T} \sum_{i=1}^{m_j} \lambda_{ij} Z_{ij}(s_i; t_j) \tag{1}$$

The multiplier λ_{ij} represents the inverse density weight (Eq. 2), which is a function of distance in space-time ($d_{ij,0}$) from the observed data $Z(s_i, t_j)$ and the space-time location of interest $\hat{Z}(s_0, t_0)$.

$$\lambda_{ij} = \frac{d_{ij,0}^{-p}}{\sum_{i=1}^{n} d_{ij,0}^{-p}} \tag{2}$$

Distance in this setting was estimated using Equation 3.

$$d((s_i, t_j), (s_0, t_0)) = \sqrt{(x_i - x_0)^2 + (y_i - y_0)^2 + C \cdot (t_j - t_0)^2} \tag{3}$$

A grid search minimising the cross-validation out-of-sample root mean square prediction error (RMSPE) on a random sample of fourteen months worth of trap data determined the values for nuisance parameters $m_j = 7$ (number of nearest neighbours), $C = 1$ (a scalar denoting the relative importance of time to geographical distance), and $p = 2$ (the inverse distance weighting factor), which corresponded to an out-of-sample RMSPE = 0.11.

The performance of the interpolation model was further evaluated based on the true and interpolated out-of-sample stabilised trap-level proportions of *w*Mel, summarised in Fig. S3. The average absolute error between the true and interpolated proportions was 0.064 (Fig. S3a). When there was error between the interpolated and observed values, the interpolated proportion tended to overestimate the observed proportion at lower values and underestimate at higher values (Fig. S3b). However, when categorised into the same categories as was ultimately used for WEI, 81.3% of out-of-sample interpolated trap proportions fell into the same category as the observed trap proportion..

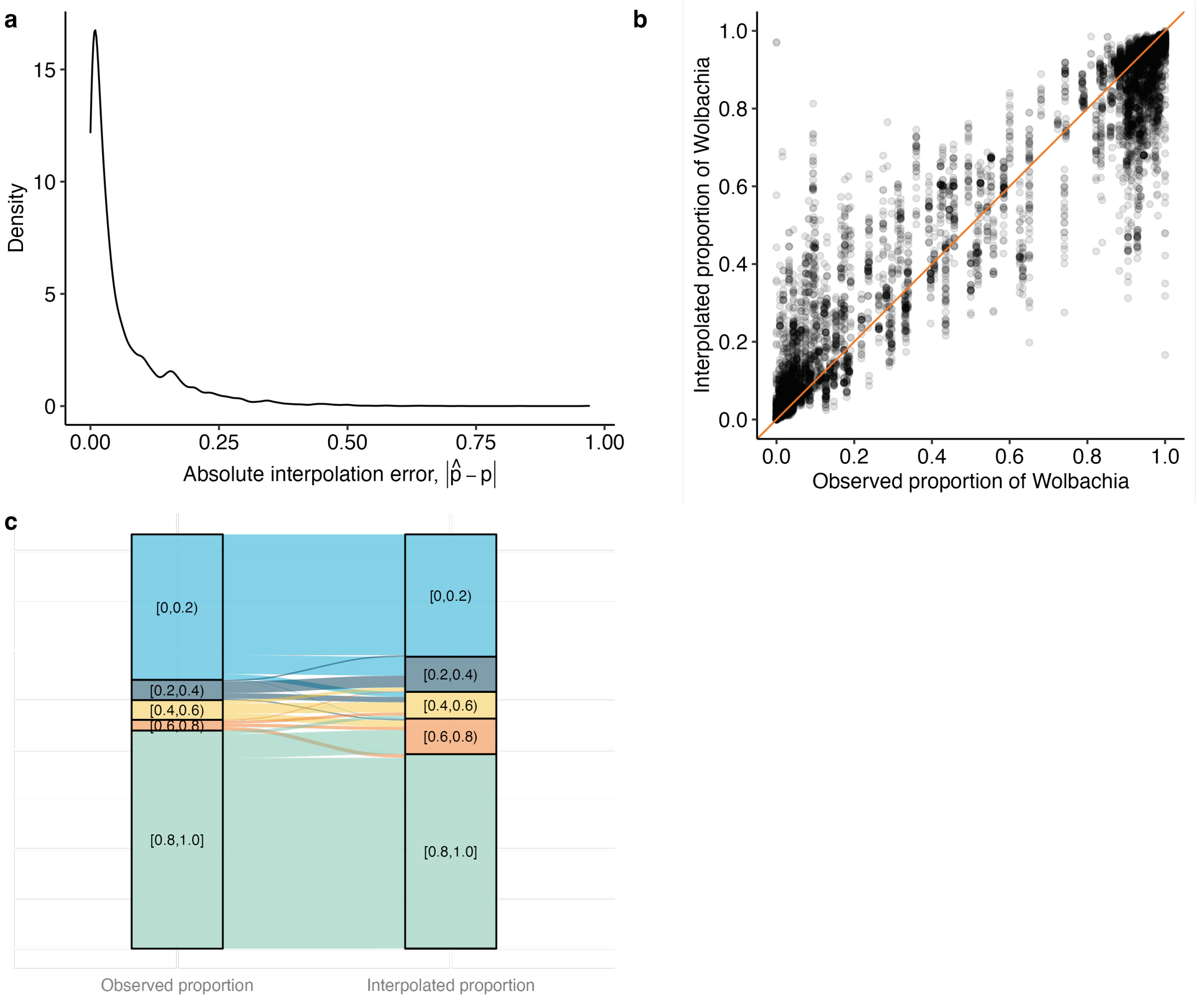

Figure S3: Visualizations of the performance of the Wolbachia spatiotemporal inverse density weighting (IDW) interpolation model, based on out-of-sample trap data. All observed trap proportions are based on the samples collected at the t and t-1 trap events. a) The absolute interpolation error for out-of-sample traps based on the observed trap wMel proportion and the interpolated trap wMel proportion from the IDW model. b) The correspondence between the out-of-sample observed wMel trap proportions and the interpolated wMel trap proportions. The red line denotes a perfect interpolation. c) The change at the categorical level used for analysis between the observed wMel trap proportion and the interpolated wMel trap proportion.

The primary sites of interpolation error (Fig. S4) occur along the borders of Intervention and Untreated clusters.

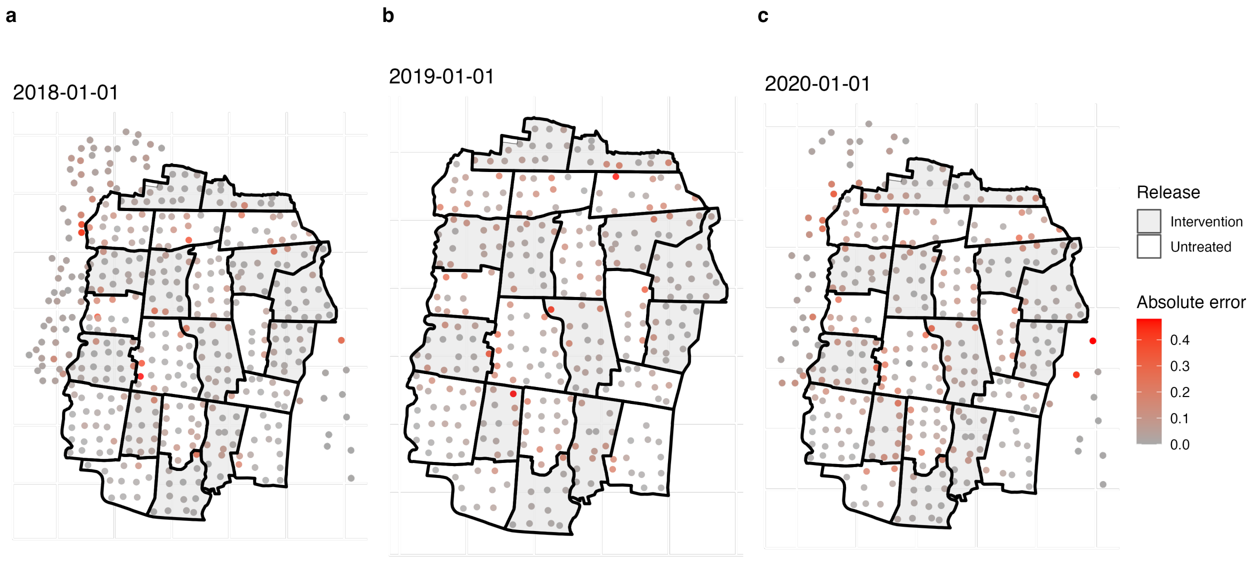

Figure S4: The absolute interpolation error for out-of-sample estimates of the trap-level proportion of wMel mosquitoes in January of a) 2018, b) 2019, and c) 2020.

3 Estimates of WEI

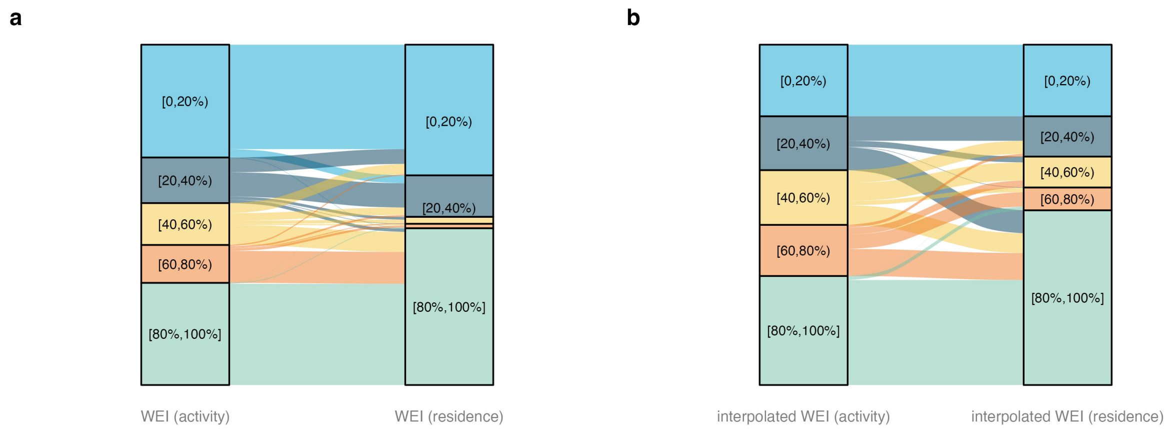

Figure S5: Comparison of participant-level WEI values based only on residential location versus time-weighted average of residential and non-residential locations. **a)** The original per-protocol individual-level WEI (activity) values (left) are compared to the original per-protocol individual-level WEI (residence) values. **b)** The individual-level WEI (activity) values from the interpolated surface (left) are compared to the individual-level WEI (residence) values from the interpolated surface (right).

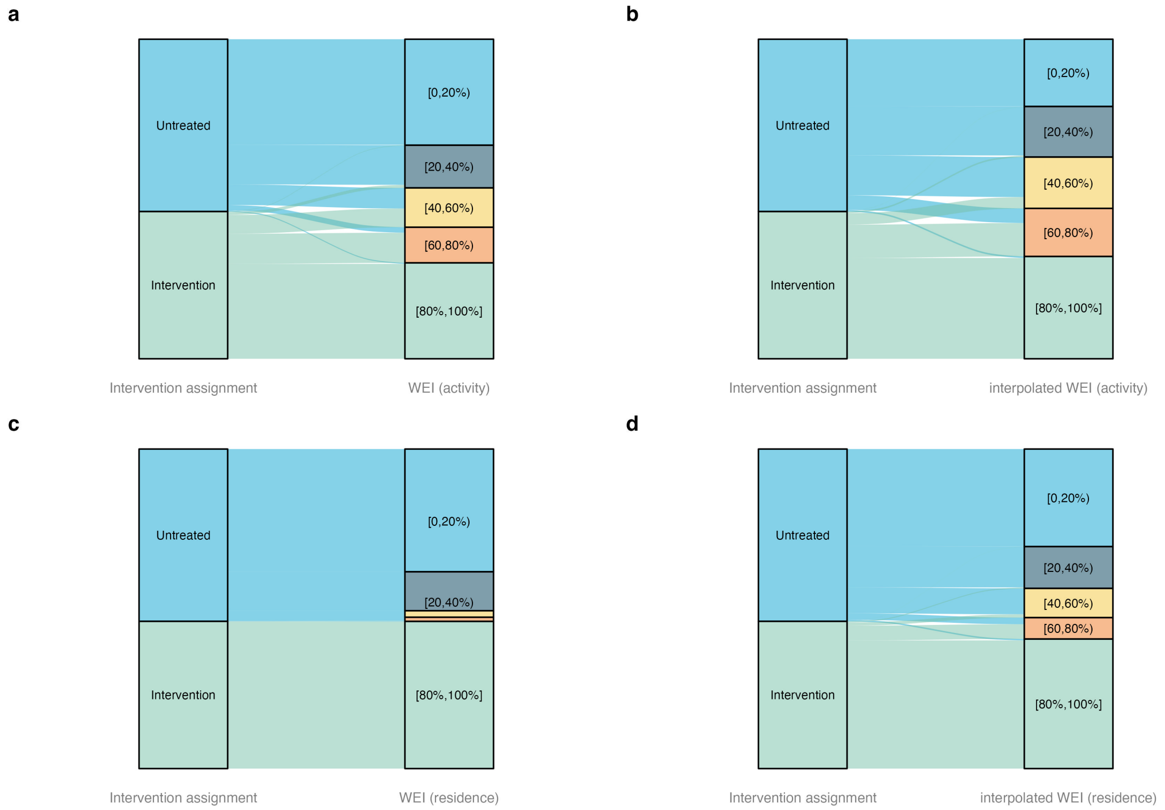

Figure S6: Changes in participant-level exposure status when based on the intervention assignment of their cluster of residence versus the various WEI methods of estimation: **a)** the original per-protocol individual-level WEI (activity) values, **b)** individual-level WEI (activity) values from the interpolated surface **c)** the original per-protocol individual-level WEI (residence) values, and **d)** the individual-level WEI (residence) values from the interpolated surface.

4 Determining the "Compliers" for Complier-Restricted Analysis

The complier-restricted analysis restricted the analytic dataset to those who spent all reported time under the intervention assignment determined by their cluster of residence (n = 3,114). Time spent outside of the AWED study area was handled two ways. First, individuals who left the AWED trial area were excluded. Second, the analysis was broadened to include individuals whose time inside and outside of the AWED study area fell under their assigned intervention arm. For individuals in the intervention arm, this means including those who only spent time in the AWED intervention area and the quasi-experiment intervention area. For those in the untreated arm, this includes individuals who spent time anywhere outside of the AWED study area except for in the quasi-experiment intervention area.
